# Molecular Epidemiology, Risk Factors and Clinical Outcomes of Carbapenem-Nonsusceptible *Enterobacter cloacae* Complex Infections in a Taiwan University Hospital

**DOI:** 10.3390/pathogens11020151

**Published:** 2022-01-25

**Authors:** Chao-Ju Chen, Po-Liang Lu, Shu-Huei Jian, Hsiao-Ling Fu, Po-Hao Huang, Chung-Yu Chang

**Affiliations:** 1 Department of Laboratory Medicine, Kaohsiung Medical University Hospital, Kaohsiung Medical University, Kaohsiung 80756, Taiwan; chaoju.chen@gmail.com; 2 College of Medicine, Kaohsiung Medical University, Kaohsiung 80708, Taiwan; d830166@gmail.com; 3 Division of Infectious Diseases, Department of Internal Medicine, Kaohsiung Medical University Hospital, Kaohsiung Medical University, Kaohsiung 80708, Taiwan; cathy30353035@gmail.com; 4 M.Sc. Program in Tropical Medicine, College of Medicine, Kaohsiung Medical University, Kaohsiung 80708, Taiwan; judy751228@yahoo.com.tw; 5 Department of Laboratory Medicine, Kaohsiung Municipal Feng-Shan Hospital (Under the Management of Chang Gung Memorail Hospital), Kaohsiung 83062, Taiwan; 6 Graduate Institute of Medicine, College of Medicine, Kaohsiung Medical University, Kaohsiung 80708, Taiwan; 990321KMUH@gmail.com; 7 Department of Microbiology and Immunology, School of Medicine, College of Medicine, Kaohsiung Medical University, Kaohsiung 80708, Taiwan; 8 School of Post-Baccalaureate Medicine, College of Medicine, Kaohsiung Medical University, Kaohsiung 80708, Taiwan

**Keywords:** *Enterobacter cloacae* complex, carbapenem, carbapenemase-producing *Enterobacteriaceae*, molecular epidemiology

## Abstract

The genus *Enterobacter* is a member of the ESKAPE group, which contains the major resistant bacterial pathogens. *Enterobacter cloacae* complex (ECC) has emerged as a clinically significant cause of a wide variety of nosocomial infections. Carbapenem-nonsusceptible *Enterobacter cloacae* complex (CnsECC) has become an emerging threat to public health but there is still a lack of comprehensive molecular and clinical epidemiological analysis. A total of 157 CnsECC isolates were recovered during October 2011 to August 2017. *hsp60* gene sequencing and pulsed-field gel electrophoresis (PFGE) were applied to discriminate the species, genetic clusters and clonal relatedness. All the isolates were subjected to polymerase chain reaction (PCR) analysis for carbapenemase, AmpC-type β-lactamase, and extended spectrum β-lactamase (ESBL) genes. Clinical data were collected on all patients for comparing clinical risks and outcomes between patients with carbapenemase-producing (CP)-CnsECC compared with non-CP-CnsECC infection. The most commonly identified species was *E. hormaechei* subsp. *hoffmannii* (47.1%), followed by *E. hormaechei* subsp. *steigerwaltii* (24.8%). Different species of CnsECC isolates showed heterogeneity in resistance patterns to piperacillin/tazobactam, cefepime and levofloxacin. In the present study, we observed that *E. hormaechei* subsp. *hoffmannii* was characterized with higher cefepime and levofloxacin resistance rate but lower piperacillin/tazobactam resistance rate relative to other species of CnsECC. CP-CnsECC comprised 41.1% (65 isolates) and all of these isolates carried IMP-8. In this study, 98% of patients had antimicrobial therapy prior to culture, with a total of 57/150 (38%) patients being exposed to carbapenems. Chronic pulmonary disease (OR: 2.51, 95% CI: 1.25–5.06), received ventilator support (OR: 5.54, 95% CI: 2.25–12.03), steroid exposure (OR: 3.88, 95% CI: 1.91–7.88) and carbapenems exposure (OR: 2.17, 95% CI: 1.10–4.25) were considered risk factors associated with CP-CnsECC infection. The results suggest that CP-CnsECC are associated with poorer outcomes including in-hospital mortality, 30-day mortality and 100-day mortality. Our study provides insights into the epidemic potential of IMP-8-producing *E. cloacae* for healthcare-associated infections and underscores the importance of understanding underlying resistance mechanisms of CnsECC to direct antibiotic treatment decisions.

## 1. Introduction

The *Enterobacter cloacae* complex (ECC) is a group of *Enterobacteriaceae* widely distributed in nature [1,2]. Currently, ECC has emerged as a clinically significant cause of a wide variety of nosocomial infections, such as pneumonia, urinary tract infections, intraabdominal infection, and bacteremia [3,4,5]. Although ECC is composed of multiple species, including *E. cloacae*, *E. hormaechei*, *E. asburiae*, *E. kobei*, *E. ludwigii*, *E. nimipressuralis* and *E. mori* [6], the routine identification of bacteria in clinical microbiology laboratories is not able to differentiate the species in the ECC by using commercialized systems [7,8]. Molecular and genomic techniques are needed to identify the ECC species precisely. Sequencing of the heat shock protein 60 (*hsp60*) gene, which can discriminate of this complex into 13 genetic clusters (C-I to CXIII) appears to be a valuable tool to identify of ECC species [3]. Furthermore, multilocus sequence analysis (MLSA) of housekeeping genes was also strongly validated by whole genome sequence (WGS) for phylogenetic classification of *Enterobacter* species [9]. Despite the fact that misidentification of ECC species has little impact on antibiotic therapy previously, as the species of the ECC have the similar antibiotic resistance profiles, these molecular approaches still have been used recently for precise identification of the species in the ECC for understanding the epidemiology, pathogenesis, and microbiological features [10].

Because of the extended overuse of antibiotics, multidrug resistant (MDR) ECC strains have emerged and spread globally. The genus Enterobacter is a member of the ESKAPE group, which are described as the major resistant bacterial pathogens and leading cause of nosocomial infections throughout the world [11]. The undesirable antibiotic resistance, especially carbapenem-resistant *Enterobacteriaceae* (CRE), is a major public health crisis because these agents are regarded as one of the last effective therapies available for treating serious infections caused by extended-spectrumβ-lactamase (ESBL)- or AmpC-producing *Enterobacteriaceae* [12]. Infections caused by CRE can lead to severe clinical outcomes and high hospitalization cost [13]. Recent surveillance studies have displayed that *Enterobacter* spp. are often the second or third most common CRE [14,15,16,17]. Therefore, Carbapenem-nonsusceptible *Enterobacter cloacae* complex (CnsECC) may become an emerging threat to public health. 

In Taiwan, Lai et al. reported that *E. cloacae* has the highest rates of carbapenem-insusceptibility among *Enterobacteriaceae* species from patients admitted to intensive care units (ICUs) in 2016 [18]. The results from Study for Monitoring Antimicrobial Resistance Trends in Taiwan (SMART) between 2016 and 2018 found that isolates of *Enterobacter* species showed higher rates of nonsusceptibility to ertapenem than *E. coli* or *K. pneumoniae* isolates [4]. The production of carbapenemases is the most important causes of carbapenem resistance in CRE [17]. The most common genes encoding carbapenemases are the *Klebsiella pneumoniae* carbapenemases (KPCs), imipenemase metallo-β-lactamases (IMPs), Verona integron encoded metallo-β-lactamases (VIMs), New Delhi metallo-β-lactamase (NDMs), and oxacillin (OXA) 48-like enzymes [19]. Global surveillance showed diversity of genes encoding carbapenemases in CnsECC [7,17]. In Taiwan, IMP type carbapenemase is highly prevalent in CnsECC [20,21,22,23,24]. IMP type carbapenemases were first discovered in Japan during the 1990s and have up to 18 varieties [19]. IMP-8 was the most common carbapenemase in *E. cloacae* from Taiwan [23]. However, comprehensive data regarding the species diversity, microbiological features, resistance plasmid characteristics and clinical relevance of CnsECC remains limited. This study was conducted to understand the epidemiology of species, clonal relatedness, carbapenemase-producing genes, and clinical features of CnsECC in Taiwan.

## 2. Results

### 2.1. Species Identification of CnsECC Isolates

**Figure 1:**
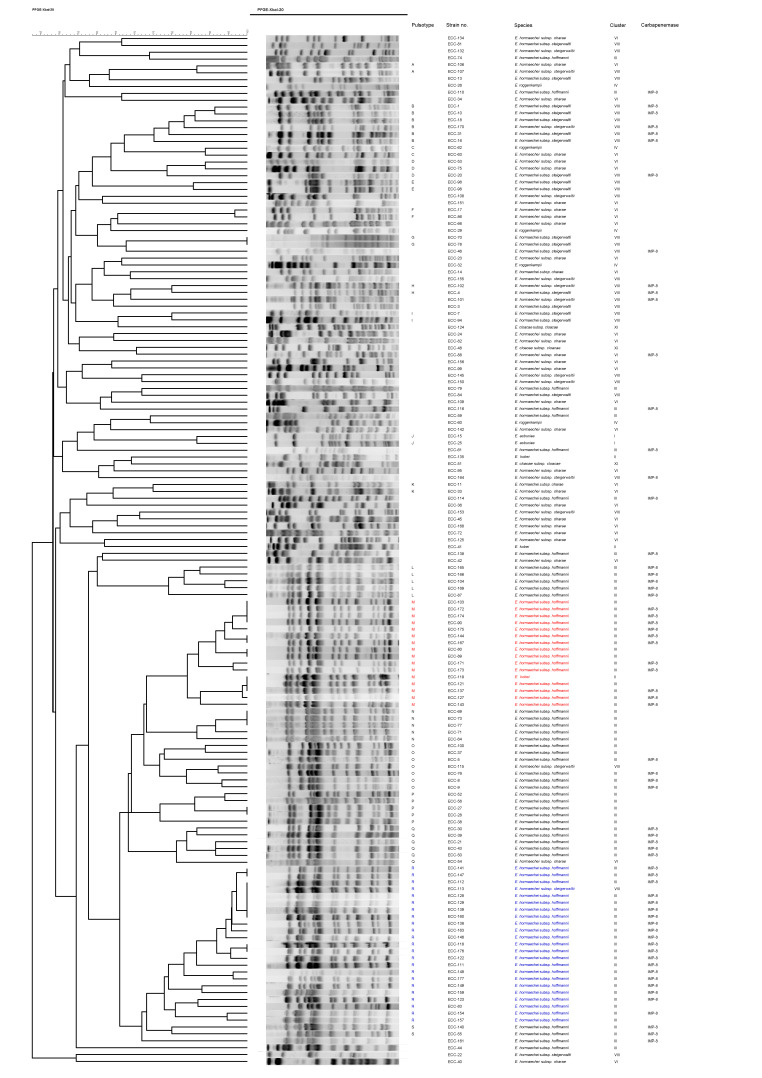
Dendrogram of pulsed-field gel electrophoresis (PFGE) cluster analysis of 150 carbapenem-nonsusceptible *Enterobacter cloacae* complex isolates. The two predominant patterns Pulsotype R (23 isolates) and Pulsotype M (16 isolates) are marked with blue and red color.

A total of 157 isolates were collected with initial reports as CnsECC from the Kaohsiung Medical University Hospital from October 2011 to August 2017. To identify species of the ECCs, we used *hsp60* gene sequencing to classify the CnsECCs into seven species (clusters) (Table 1). The most commonly identified species was *E. hormaechei* subsp. *hoffmannii* (cluster III) (74/157, 47.1%), followed by *E. hormaechei* subsp. *steigerwaltii* (cluster VIII) (39/157, 24.8%), *E. hormaechei* subsp. *oharae* (cluster VI) (30/157, 19.1%), *E. roggenkampii* (cluster IV) (6/157, 3.8%), *E. kobei* (cluster II) (3/157, 1.9%), *E. cloacae* subsp. *cloacae* (cluster XI) (3/157, 1.9%) and *E. asburiae* (cluster I) (2/157, 1.3%). For further discrimination, pulsed-field gel electrophoresis (PFGE) analysis was performed. Among the 157 CnsECC isolates, 150 isolates were successfully obtained the PFGE banding results (Figure 1). In PFGE analysis, 96 of 150 isolates (64%) can be clustered into 19 pulsotypes (A-S) with >75% similarity in PFGE banding patterns and the two predominant patterns were Pulsotype R (23 isolates) and Pulsotype M (16 isolates). In these two pulsotypes, 22 and 15 isolates belonged to *E. hormaechei* subsp. *hoffmannii*, respectively.

### 2.2. Antimicrobial Susceptibility of CnsECC Isolates

**Figure 2:**
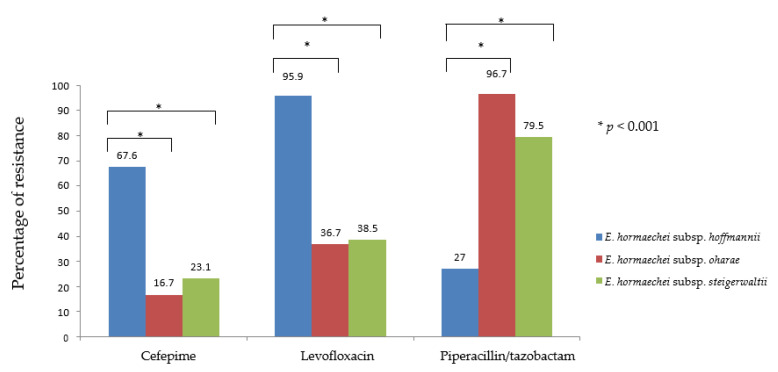
The statistical analysis among three predominant species *(E. hormaechei* subsp. *hoffmannii*, *E. hormaechei* subsp. *oharae* and *E. hormaechei* subsp. *steigerwaltii*) in cefepime, levofloxacin and piperacillin/tazobactam.

All of the 157 CnsECC isolates were resistant to multiple antimicrobial agents, such as cefazolin, cefmetazole, and ampicillin. Most of the isolates were resistant to ampicillin-sulbactam, ceftazidime, sulfamethoxazole-trimethoprim and levofloxacin. Almost all of the isolates remain susceptible to amikacin (99.4%). The antimicrobial susceptibility testing is summarized in Table 2. Notably, the prevalence of resistance was significantly different among three predominant species (*E. hormaechei* subsp. *hoffmannii*, *E. hormaechei* subsp. *oharae* and *E. hormaechei* subsp. *steigerwaltii*) in cefepime, levofloxacin and piperacillin/tazobactam (Table 3 and Figure 2). The cefepime resistance was higher in *E. hormaechei* subsp. *Hoffmannii* (67.6%) than in *E. hormaechei* subsp. *oharae* and *E. hormaechei* subsp. *steigerwaltii* (16.7% and 23.1%) respectively (*p* < 0.001). The overall resistant rate to levofloxacin was 65.6% and was significantly higher in *E. hormaechei* subsp. *hoffmannii* (95.9%) and lower in *E. hormaechei* subsp. *oharae* (36.7%) and *E. hormaechei* subsp. *steigerwaltii* (38.5%) (*p* < 0.001). In contrast, resistance to piperacillin/tazobactam was 58.0% overall, but was significantly lower in *E. hormaechei* subsp. *hoffmannii* (27.0%) and higher in *E. hormaechei* subsp. *oharae* (96.7%) and *E. hormaechei* subsp. *steigerwaltii* (79.5%) (*p* < 0.001) (Table 3 and Figure 2).

### 2.3. Detection of Genes Encoding Carbapenemases, AmpC and ESBLs in CnsECC Isolates

The 157 CnsECC isolates were examined for the presence of genes encoding the Carbapenemase (*bla*_carbapenemase_), ESBL (*bla*_ESBL_) and AmpC (*bla*_AmpC_), which is demonstrated in Table 4. Carbapenemase-producing Enterobacteriaceae comprised 41.4% (65 isolates) of the 157 CnsECC isolates and all of them were positive for IMP-8. Among 65 IMP-8-positive CnsECC isolates, 81.5% (53 isolates) belonged to *E. hormaechei* subsp. *hoffmannii* (cluster III), 16.9% (11 isolates) were *E. hormaechei* subsp. *steigerwaltii* (cluster VIII) and 1.5% (1 isolate) was *E. hormaechei* subsp. *oharae* (cluster VI). There were 33 (21%) isolates carried ESBL genes and the predominant *bla*_ESBL_ was SHV-12 (31 isolated, 94%). While 59 (37.6%) isolates were *bla*_AmpC_-positive, and genes encoding ACT, CMY, DHA-1 and MIR accounted for 78% (46 isolates), 5% (2 isolates), 8% (5 isolates) and 10% (6 isolates) of the *bla*_AmpC_-positive CnsECC isolates. It is worth nothing that coexistence of ESBL and AmpC, and the carriage of multiple ESBL and/or AmpC was common, especially in *E. hormaechei* subsp. *hoffmannii* (cluster III). Cocarriage of IMP-8 and SHV-12 was detected in eight isolates and cocarriage of IMP-8 and ACT was detected in 24 isolates, respectively. Besides, coexistence of IMP-8, SHV-12 and ACT was noted in four isolates of CnsECC (Table 4).

### 2.4. Carbapenem MIC Distribution of CnsECC Isolates with and without IMP-8

IMP-8 is the only carbapenemase-encoding gene detected in this study. To evaluate possible effects of IMP-8 on the carbapenems MIC, we conducted antimicrobial susceptibility testing of ertapenem and meropenem. As shown in Table 5, regardless of harboring a carbapenemase-encoding gene, almost all (99.4%) the CnsECC strains were resistant to ertapenem (MIC50 = 4 μg/ mL). In contrast, in 65 IMP-8-positive strains, 35 (53.8%) remained susceptible to meropenem and among the 92 non-carbapenemase producing CnsECC (non-CP-CnsECC) strains, most of them (86 isolates, 93.5%) were susceptible to meropenem.

### 2.5. Clinical Features and Data Analysis

Between October 2011 and August 2017, a total of 150 patients with nonduplicate CnsECC isolates were identified. Baseline characteristics and prior health care exposure of the patients with CnsECC are shown in Table 6. Their median age was 68 years, 62 (41.3%) were female, and 64 (42.7%) were carbapenemase-producing *Enterobacter cloacae* complex (CP-ECC) carriers. Almost all individuals had underlying comorbid conditions; the majority of patients had underlying cardiovascular disease (*n* = 105, 70%), followed by chronic kidney disease (*n* = 94, 62.7%), chronic pulmonary disease (*n* = 92, 61.3%), diabetes mellitus (*n* = 65, 43.3%), chronic liver disease (*n* = 62, 41.3%), cerebrovascular disease (*n* = 49, 32.7%), hematological malignancies (*n* = 48, 32.0%), and solid tumor (*n* = 46, 30.7%). Most of the patients with CnsECC had catheter devices indwelling (*n* = 138, 92%), ventilator support (*n* = 93, 62%) and antibiotics exposure (*n* = 147, 98%) within 3 months prior to isolation of CnsECC. The average length of days from admission to positive CnsECC culture was 26 days (IQR: 5.25–33). In the statistics analysis for CP-ECC acquisition compared with CnsECC without carbapenemase production (non-CP-ECC), significantly greater chronic pulmonary disease (OR: 2.51; 95% CI: 1.25–5.06), ventilator support (OR: 5.54; 95% CI: 2.55–12.03) and carbapenem exposure (OR: 2.17; 95% CI: 1.10–4.25) were associated with the CP-ECC group.

### 2.6. Culture Sources and Outcomes of Patients with CnsECC Infection

The distributions of the culture source and outcome of CnsECC cases are shown in Table 7. A total of 150 consecutive nonduplicate CnsECC isolates were originated from different anatomical sites: blood (*n* = 15, 10.0%), sputum (*n* = 49, 32.7%), urine (*n* = 51, 34.0%), pus (*n* = 21, 14.0%), body fluid (*n* = 7, 4.6%), catheter (*n* = 2, 1.3%), catheter (*n* = 1, 3.4%), bronchoalveolar lavage (*n* = 3, 2.0%), and swab (*n* = 2, 1.3%). The incidence was no different in each culture source between CP-ECC and non-CP-ECC. The average length of stay for all CnsECC patients was 37.5 days (IQR, 20–64). There was no difference in length of hospitalization, between CP-ECC and non-CP-ECC. Patients with CP-ECC infection were more likely to have a higher in-hospital mortality (OR: 2.6; 95% CI: 1.25–5.40), 30-day mortality (OR: 3.01; 95% CI: 1.34–6.72) and 100-day mortality (OR: 2.84; 95% CI: 1.34–6.03) compared with non-CP-ECC.

## 3. Discussion

An increasing incidence of CnsECC poses a great threat to public health [7,25]. There, it is urgent to characterize the clinical molecular epidemiology of CnsECC infection. In this study, we demonstrated that the most common species of CnsECC in southern Taiwan was *E. hormaechei* subsp. *hoffmannii* (cluster III) (47.1%), which is similar to previous study for ECC isolates co-resistant to carbapenem and colistin in southeast China [3]. The second common species of CnsECC in this study was *E. hormaechei* subsp. *steigerwaltii* (cluster VIII) (24.8%), which was the predominant strain in northeast China (38.9%) [17] and Japan (33.3%) [26]. 

It is well known that ECC has an intrinsic resistance to ampicillin, amoxicillin and first- and second-generation cephalosporins owing to low expression of chromosomal *ampC* gene encoding an inducible AmpC β-lactamase under a basal condition [7,11,27]. Expectedly, most of the CnsECC isolates in the present study showed resistant to ampicillin, ampicillin/sulbactam, and first- and second-generation cephalosporins. It is noteworthy that the different species of CnsECC isolates showed heterogeneity in resistance patterns to cefepime, levofloxacin and piperacillin/tazobactam. Our data demonstrated *E. hormaechei* subsp. *hoffmannii* (cluster III) was characterized with higher cefepime and levofloxacin resistance rate but lower piperacillin/tazobactam resistance rate relative to other species of CnsECC. A similar pattern of results was also revealed in a recent study, which reported different antibiotics resistant pattern between two identical genetic clusters among carbapenem-resistant ECC isolates [17]. These observations indicate multiple drug-resistant mechanisms may participate in CnsECC isolates and warrant further investigation.

The resistant mechanisms of CRE are the presence of either carbapenemase or ESBLs and/or AmpC enzyme as well as losing outer membrane porin proteins [23,28,29]. Our study showed the predominant mechanism of CnsECC isolates were carbapenemase production (41.4%) and AmpC overproduction (37.3%). This result is compatible with previous studies demonstrating carbapenemase production and overproduction of AmpC are the main carbapenem resistance mechanisms in CRE isolates [28,29,30]. The prevalence of CP-CnsECC among clinical CnsECC isolates was around 41.4% (65/157 isolates) and IMP-8 was the only carbapenemase detected in the CP-ECC isolates (65/65 isolates, 100%) in this study. IMP-encoding ECC have been reported globally, including in China (IMP-1 and IMP-34), Thailand (IMP-14), Japan (IMP-1 and IMP-11), Australia (IMP-4) and Korea (IMP-4) [31]. In Taiwan, IMP-8-encoding CnsECC has been reported since the early 2000s [24]. In a large surveillance report for carbapenem-nonsusceptible Enterobacteriaceae during 2010–2012 in Taiwan observed 96.3% of CP-CnsECC had IMP-8 which agrees with our findings [23]. 

In the present study, we defined CnsECC as ECC isolates with nonsusceptibility to ertapenem. Among the 157 CnsECC isolates, we observed that isolates tests showing susceptibility to meropenem were common (77.1%, 121/157). Similar results have been found by other studied [23,24,32]. Meanwhile, we found almost all non-CP-CnsECC were susceptible to meropenem (93.5%, 86/92) and lower meropenem susceptibility rates in CP-CnsECC isolates (53.8%, 35/65). This finding is also correlated with previous studies for carbapenem-nonsusceptible *Klebsiella pneumoniae* (CnsKP), which showed CP-CnsKP was more nonsusceptible to meropenem than non-CP-CnsKP [33]. Consequently, non-CP-CnsECC might be effectively treated by meropenem in current circumstances. Aside from the carbapenem, several novel antibiotics, such as ceftazidime-avibactam, ceftozolane-tazobactam, cefepime-zidebactam, meropenem-vaborbactam, cefiderocol and eravacycline have been developed to treat multidrug-resistant organisms but many of them have limited activity against strains bearing class B metallo-lactamases (MBLs) such as IMP. Therefore, using novel rapid molecular/ lateral flow infectious disease diagnostic tools such as Xpert^®^ Carba-R test (Cepheid, Sunnyvale, CA, USA), Biofire FilmArray panels (Biomerieux, Nürtingen, Germany) or NG-Test Carba 5 (NG Biotech, Guipry, France) to identify the type of carbapenemase present in the carbapenem nonsusceptible isolate is important for optimizing antibiotics therapy [34,35].

In this study, 98% of patients had antimicrobial therapy prior to culture, with a total of 57/150 (38%) patients being exposed to carbapenems. Chronic pulmonary disease and carbapenems exposure were considered risk factors associated with CP-CnsECC. Besides, we observed CP-CnsECC associated with poorer outcomes including in-hospital mortality, 30-day mortality and 100-day mortality. A previous study demonstrated CP-CRE may be more virulent than non-CP-CRE and is associated with poorer outcomes [36]. However, several studies showed there was no difference in clinical outcomes [25,26,37]. The heterogeneity could be related to various carbapenemase-producing genes in CRE were predominant in different species of Enterobacteriaceae or geographic regions and it highlights the need to understand the local epidemiology to tailor prevention efforts in individual regions. This is the first study to make comparison of clinical characteristics and patient outcomes between carbapenemase-producing and non-carbapenemase-producing CnsECC isolates in Taiwan.

This study has several limitations. Due to the lack of available medical information for ascertainment of infections, our data likely overestimated the proportion of lower respiratory and urinary tract infections. Furthermore, we did not have a control group of carbapenem-susceptible ECC. Therefore, we were not able to conduct risk factor analysis on CnsECC. There is also the possibility that novel carbapenemases or rarer enzymes that were not performed. Finally, as in any observational study, information on clinical characteristics and outcomes could not be completely acquired and our analysis of clinical outcome was subject to confounding biases.

## 4. Materials and Methods

### 4.1. Bacterial Isolates

A total of 157 ertapenem-nonsusceptible *Enterobacter cloacae* complex isolates (minimum inhibitory concentrations [MICs] of ertapenem >1 μg/mL) were collected from Kaohsiung Medical University Hospital during October 2011 to August 2017 and defined as CnsECC. The identification of ECC and testing of ertapenem susceptibility were performed as routine clinical microbiology laboratory procedures. The collection and testing of clinical specimens were approved by the Review Board Committee of KMUH.

### 4.2. Antimicrobial Susceptibility Testing

Antimicrobial susceptibility was tested by the broth dilution method according to the guidelines of the Clinical and Laboratory Standards Institute (CLSI) [38]. All isolates were tested for minimal inhibitory concentrations (MICs) of β-lactam agents, including penicillins (ampicillin, ampicillin/sulbactam, piperacillin-tazobactam), cephalosporins (cefazolin, cefmetazole, ceftazidime and cefepime), carbapenems (ertapenem and meropenem); and non-β-lactams, including fluoroquinolone (levofloxacin), aminoglycosides (gentamicin and amikacin), trimethoprim-sulfamethoxazole and tigecycline.

### 4.3. Amplification and Sequencing of the hsp60 Gene

Polymerase chain reaction (PCR) analysis for partial sequencing of the *hsp60* gene was performed by a protocol described previously [39]. A 341-bp fragment of the *hsp60* gene was amplified and sequencing. The sequences of a 272-bp fragment of the *hsp60* gene obtained for 157 strains were analyzed by the nucleotide BLAST program searches against the NCBI database and each isolate was assigned to its respective species, subspecies, and cluster according to taxonomic studies published previously [6,39].

### 4.4. Pulsed Field Gel Electrophoresis

Pulsed-field gel electrophoresis (PFGE) XbaI (New England Biolabs, Beverly, MA, USA)-digested genomic DNA was conducted to delineate the genetic relatedness of the isolates using procedures described previously [40]. PFGE patterns were interpreted in accordance with the criteria of Tenover et al. [41]. Restriction fragments were analyzed using GelCompar II software 6.5 (Applied Maths, Austin, TX, USA), and dendrograms of the patterns were constructed using the unweighted pair group method with arithmetic mean (UPGMA) based on the Dice similarity index.

### 4.5. Detection of Genes Encoding Carbapenemases, AmpC, and ESBLs

All verified isolates were subjected to polymerase chain reaction (PCR) detection of genes encoding carbapenemases (IMP, KPC, OXA, NDM, VIM, BIC, IMI, SME, AIM, DIM, GIM, SPM, SIM, and GES) [42], AmpC genes (CMY, DHA, and ACT) [43] and ESBL genes (CTX-M, SHV, and TEM) [44].

### 4.6. Clinical Data Collection and Statistical Analyses

This was a single-center, retrospective, observational study of patients with positive cultures of CnsECC from October 2011 to August 2017 at KUMH. Patient information was retrospectively retrieved via electronic medical records. The parameters included demographic data, comorbidities, healthcare exposures (such as indwelling devices, hemodialysis, mechanical ventilation, and surgeries), exposure to antimicrobials within 3 months prior to isolation of CnsECC, and the clinical outcomes. Clinical outcomes were assessed by either in-hospital mortality or patient survival. The Student *t*-test was used for continuous variables. The chi-square test or Fisher exact test was used to compare categorical variables. For categorical variables with more than two categories, we used ordinary logistic regression to perform multiple comparisons. Statistical significance was set at *p* < 0.05. All statistical analyses were performed using MedCalc statistical software version 20.013 (MedCalc Software Corporation, Ostend, Belgium).

## 5. Conclusions

In conclusion, nearly all the CnsECC isolates in the present study were resistant to ampicillin and first- and second-generation cephalosporins but the majority remained susceptible to amikacin and meropenem. Different species of CnsECC isolates showed heterogeneity in resistance patterns to cefepime, levofloxacin and piperacillin/tazobactam. The most common species of CnsECC was *E. hormaechei* subsp. *Hoffmannii,* which was characterized with higher cefepime and levofloxacin resistance rate and with higher prevalence of CP-CnsECC. IMP-8 was the only carbapenemases detected among the CnsECC isolates in this study. Analysis on clinical data revealed patient with CP-CnsECC infection had poorer clinical outcomes. This study highlights the need to understand the local molecular epidemiology of carbapenem-nonsusceptible ECC and shows that using novel rapid infectious disease diagnostic tools to identify the type of carbapenemase is important for optimizing antibiotics therapy against CnsECC.

## Figures and Tables

**Table 1:** Species identification by *hsp60* gene sequencing and PFGE pulsotypes of CnsECC isolates.

Species	Cluster	No. of Isolates (%)	PFGE Pulsotype (*n*)
*Enterobacter asburiae*	I	2 (1.3)	J (2)
*Enterobacter kobei*	II	3 (1.9)	M (1)
*Enterobacter hormaechei* subsp. *hoffmannii*	III	74 (47.1)	L (5) M (15) N (5) O (6) P (5) Q (5) R (22) S (2)
*Enterobacter roggenkampii*	IV	6 (3.8)	C (1)
*Enterobacter hormaechei* subsp. *oharae*	VI	30 (19.1)	A (1) C (1) D (2) F (2) K (2) Q (1)
*Enterobacter hormaechei* subsp. *steigerwaltii*	VIII	39 (24.8)	A (1) B (6) D (1) E (2) G (2) H (2) I (2) O (1) R (1)
*Enterobacter cloacae* subsp. *cloacae*	XI	3 (1.9)	
Total		157	96

**Table 2:** Antimicrobial susceptibility of CnsECC isolates ^a^.

Antimicrobial Agents	R		I/SDD ^b^		S		MIC (μg/mL)		
	*n*	%	*n*	%	*n*	%	MIC50	MIC90	Range
Ampicillin	157	100.0	0	0	0	0	-	-	-
Amikacin	1	0.6	0	0	156	99.4	≤2	16	≤2–≥64
Ceftazidime	148	94.3	2	1.3	7	4.5	≥64	≥64	2–≥64
Cefmetazole	157	100.0	0	0	0	0	≥64	≥64	≥64
Cefazolin	157	100.0	0	0	0	0	≥64	≥64	≥64
Ertapenem	156	99.4	1	0.6	0	0	4	≥8	1–≥8
Cefepime	65	41.4	33	21.0	59	37.6	8	≥64	≤1–≥64
Gentamicin	38	24.2	7	4.5	112	71.3	2	≥16	≤1–≥16
Levofloxacin	103	65.6	12	7.6	42	26.8	≥8	≥8	≤0.12–≥8
Meropenem	31	19.7	5	3.2	121	77.1	0.5	8	≤0.25–≥16
Ampicillin/sulbactam	156	99.4	0	0	1	0.6	-	-	-
Sulfamethoxazol/trimethoprim	113	72.0	0	0	44	28.0	≥16/304	≥16/304	≤1/19–≥16/304
Tigecycline	81	51.6	10	6.4	66	42.0	≥8	≥8	≤0.5–≥128
Piperacillin/tazobactam	91	58.0	37	23.6	29	18.5	≥128/4	≥128/4	≤4/4–≥128/4

^a^ Abbreviations: R, resistant; I, intermediate; S, susceptible; SDD, susceptible-dose dependent. ^b^ SDD for cefepime.

**Table 3:** Antimicrobial resistance in each species of CnsECC isolates.

Species (*n*) Cluster		AM		AN		CAZ		CMZ		CZ		ETP		FEP	
		*n*	%	*n*	%	*n*	%	*n*	%	*n*	%	*n*	%	*n*	%
*E. asburiae* (2)	I	2	100	0	0.0	2	100	2	100	2	100	2	100	0	0.0
*E. kobei* (3)	II	3	100	0	0.0	3	100	3	100	3	100	3	100	1	33.3
*E. hormaechei *subsp.* hoffmannii* (74)	III	74	100	0	0.0	71	95.9	74	100	74	100	74	100	50	67.6
*E. roggenkampii* (6)	IV	6	100	0	0.0	4	66.7	6	100	6	100	6	100	0	0.0
*E. hormaechei* subsp. *oharae* (30)	VI	30	100	1	3.3	28	93.3	30	100	30	100	30	100	5	16.7
*E. hormaechei* subsp. *steigerwaltii* (39)	VIII	39	100	0	0.0	38	97.4	39	100	39	100	38	97.4	9	23.1
*E. cloacae* subsp. *cloacae* (3)	XI	3	100	0	0.0	2	66.7	3	100	3	100	3	100	0	0.0
Total (157)		157	100	1	0.6	148	94.3	157	100	157	100.0	156	99.4	65	41.4
**Species (*n*) Cluster**		**GM**		**LEV**		**MEM**		**SAM**		**SXT**		**TGC**		**TZP**	
		*n*	%	*n*	%	*n*	%	*n*	%	*n*	%	*n*	%	*n*	%
*E. asburiae* (2)	I	0	0.0	2	100	0	0.0	2	100	0	0.0	0	0.0	2	100
*E. kobei* (3)	II	0	0.0	2	66.7	0	0.0	3	100	2	66.7	1	33.3	3	100
*E. hormaechei *subsp.* hoffmannii* (74)	III	18	24.3	71	95.9	24	32.4	74	100	72	97.3	52	70.3	20	27.0
*E. roggenkampii* (6)	IV	0	0.0	1	16.7	0	0.0	6	100	1	16.7	0	0.0	4	66.7
*E. hormaechei* subsp. *oharae* (30)	VI	8	26.7	11	36.7	2	6.7	30	100	15	50.0	12	40.0	29	96.7
*E. hormaechei* subsp. *steigerwaltii* (39)	VIII	12	30.8	15	38.5	5	12.8	38	97.4	22	56.4	16	41.0	31	79.5
*E. cloacae* subsp. *cloacae* (3)	XI	0	0.0	1	33.3	0	0.0	3	100	1	33.3	0	0.0	2	66.7
Total (157)		38	24.2	103	65.6	31	19.7	156	99.4	113	72.0	81	51.6	91	58.0

AM, ampicillin; AN, amikacin; CAZ, ceftazidime; CMZ, cefmetazole; CZ, cefazolin; ETP, ertapenem; FEP, cefepime; GM, gentamicin; LEV, levofloxacin; MEM, meropenem; SAM, ampicillin/sulbactam; SXT, sulfamethoxazol/trimethoprim; TGC, tigecycline; TZP, piperacillin/tazobactam.

**Table 4:** β-lactamase genes of CnsECC isolates.

		β-Lactamase Genes (*n*)				
Species (*n*)	Cluster	Carbapenemase	*bla*_ESBL_	*bla*_AmpC_	Co-Existence of *bla*_ESBL_, *bla*_AmpC_, or Carbapenemase Genes	Other β-Lactamase Genes
*E. asburiae* (2)	I			ACT (2)		
*E. kobei* (3)	II					
*E.hormaechei* subsp. *hoffmannii* (74)	III	IMP-8 (53)	SHV-12 (18) CTX-M9 (1)	CMY (2) ACT (43) DHA (1)	IMP-8 + SHV-12 + ACT (4) IMP-8 + SHV-12 (2) IMP-8 + CMY-2 + ACT (1) IMP-8 + CMY-2 (1) IMP-8 + ACT (23) SHV-12 + CTX-M9 + ACT (1) SHV-12 + ACT (9)	TEM-1 (11) OXA-1 (1)
*E. roggenkampii* (6)	IV			MIR (6)		
*E. hormaechei* subsp. *oharae* (30)	VI	IMP-8 (1)	SHV-12 (6)	ACT (1) DHA-1 (3)	SHV-12 + DHA-1 (3)	TEM-1 (13) OXA-1 (1)
*E. hormaechei* subsp. *steigerwaltii* (39)	VIII	IMP-8 (11)	SHV-12 (7) CTX-M14 (1)	DHA-1 (1)	IMP-8 + SHV-12 (6)	TEM-1 (13) OXA-1 (2)
*E. cloacae* subsp. *cloacae* (3)	XI					TEM-1 (2)
Total (157)		65 (41.4%)	33 (21.0%)	59 (37.6%)	50 (31.8%)	43 (27.4%)

**Table 5:** Carbapenem MIC distribution among CnsECC isolates with or without carbapenemase IMP-8.

IMP-8/Carbapenem	MIC (μg/mL)										
	≤0.25	0.5	1	2	4	8	≥8	≥16	MIC_50_	MIC_90_	Range
IMP-8 positive (*n* = 65)											
Ertapenem			1	2	36		26		4	≥8	1–≥8
Meropenem	8	10	17	4	9	4		13	1	≥16	≤0.25–≥16
IMP-8 negative (*n* = 92)											
Ertapenem				31	51		10		4	≥8	2–≥8
Meropenem	68	6	12	1	3	1		1	≤0.25	1	≤0.25–≥16

Gray-shaded cells indicate resistance.

**Table 6:** Baseline characteristics and health care exposure of CnsECC.

Variables	CnsECC *n* = 150	Non-CP-EnsCC *n* = 86	CP-EnsCC *n* = 64	OR (95% CI)	*p*
Sex, female	62	36	26	0.95 (0.49–1.83)	0.88
Age (years), median (range)	68 (4–95)	70.5 (4–95)	63 (34–94)		0.64
Cardiovascular disease	105 (70%)	62	43	0.79 (0.39–1.60)	0.52
Cerebrovascular disease	49 (32.7%)	26	23	1.29 (0.65–2.57)	0.46
Diabetes mellitus	65 (43.3%)	35	30	1.29 (0.67–2.47)	0.45
Chronic pulmonary disease	92 (61.3%)	45	47	2.51 (1.25–5.06)	0.01 *
Chronic liver disease	62 (41.3)	40	22	0.60 (0.31–1.17)	0.14
Chronic kidney disease	94 (62.7)	55	39	0.88 (0.45–1.71)	0.71
Solid tumor	46 (30.7)	28	18	0.81 (0.40–1.65)	0.56
Hematological malignancies	48 (32.0)	27	21	1.05 (0.65–1.67)	0.85
Health care exposure (within 3 months)					
Indwelling devices	138 (92)	78	60	1.54 (0.44–5.39)	0.50
Hemodialysis	26 (17.3)	14	12	1.18 (0.51–2.78)	0.69
Ventilator support	93 (62)	40	53	5.54 (2.55–12.03)	<0.001 *
Surgery	63 (42)	36	27	1.01 (0.53–1.96)	0.97
Chemotherapy	12 (8)	6	6	1.38 (0.42–4.49)	0.59
Steroid exposure	53 (35.8)	19	34	3.88 (1.91–7.88)	<0.001 *
Antibiotics exposure	147 (98)	83	64	5.41 (0.27–106.6)	0.27
Carbapenems exposure	57 (38)	25	31	2.17 (1.10–4.25)	0.02 *

Abbreviations: CnsECC, Carbapenem-nonsusceptible *Enterobacter cloacae* complex; CP, carbapenemase-producing. * Statistical significance, *p* < 0.05.

**Table 7:** Culture source and clinical outcome among CnsECC.

Variables	CnsECC *n* = 150	Non-CP-EnsCC *n* = 86	CP-EnsCC *n* = 64	OR (95% CI)	*p*
Culture source					0.10
Blood	15 (10.0%)	9	6	0.89 (0.30–2.63)	0.83
Sputum	49 (32.7%)	23	26	1.03 (0.52–2.04)	0.93
Urine	51 (34.0%)	29	22	1.87 (0.94–3.74)	0.07
Abscess	21 (14.0%)	15	6		
Body fluid	7 (4.6%)	6	1		
Catheter	2 (1.3%)	2	0		
BAL	3 (2.0%)	0	3		
Swab	2 (1.3%)	2	0		
Outcomes					
Length of hospitalization, (median days, IQR)	37.5 (20–64)	29.5 (17–57)	45.5 (28–64)		0.48
In-hospital mortality	42 (28)	17	25	2.60 (1.25–5.40)	0.01 *
30-day mortality	33 (22)	12	21	3.01 (1.34–6.72)	0.007 *
100-day mortality	39 (26)	15	24	2.84 (1.34–6.03)	0.007 *

Abbreviations: CnsECC, carbapenem-nonsusceptible *Enterobacter cloacae* complex; CP, carbapenemase-producing; IQR, interquartile range. * Statistical significance, *p* < 0.05.

## Data Availability

Not applicable.
